# Influence of folate status on genomic DNA methylation in colonic mucosa of subjects without colorectal adenoma or cancer

**DOI:** 10.1038/sj.bjc.6602439

**Published:** 2005-02-22

**Authors:** M Pufulete, R Al-Ghnaniem, J A Rennie, P Appleby, N Harris, S Gout, P W Emery, T A Sanders

**Affiliations:** 1 Nutritional Sciences Research Division, King's College London, Franklin Wilkins Building, 150 Stamford Street, London SE1 9NH, UK; 2 Department of Surgery, King's College Hospital, Denmark Hill, London SE5 9RS, UK; 3 Cancer Research UK Epidemiology Unit, The Radcliffe Infirmary, Oxford OX2 6HE, UK; 4 LGC Limited, Queens’ Road, Teddington, Middlesex TW11 0LY, UK

**Keywords:** folate, homocysteine, DNA methylation, colorectal cancer, genotype, MTHFR, MS, CBS

## Abstract

DNA hypomethylation may increase the risk of colorectal cancer. The main aim of this study was to assess the influence of folate status (serum and erythrocyte folate and plasma homocysteine concentrations) on DNA methylation. Methylenetetrahydrofolate reductase (MTHFR 677C → T and 1298A → C), methionine synthase (MS 2756A → G) and cystathionine synthase (CBS 844ins68) polymorphisms were measured to account for potential confounding effects on folate status and DNA methylation. A total of 68 subjects (33 men and 35 women, 36–78 years) free from colorectal polyps or cancer were recruited in a cross-sectional study. Tissue biopsies were obtained at colonoscopy for the determination of DNA methylation in colonic mucosa using an *in vitro* radiolabelled methyl acceptance assay. Serum and erythrocyte folate were inversely correlated with plasma homocysteine (*r*=−0.573, *P*<0.001 and *r*=−0.307, *P*=0.01 respectively) and DNA hypomethylation in colonic mucosa (*r*=−0.311, *P*=0.01 and *r*=−0.356, *P*=0.03). After adjusting for gender, age, body mass index, smoking and genotype, there were weak negative associations between serum and erythrocyte folate and colonic DNA hypomethylation (*P*=0.07 and *P*=0.08, respectively).

A low dietary intake of folate has been associated with increased risk of colorectal adenoma and cancer in prospective cohort (Giovannucci *et al*, 1993; Giovannucci *et al*, 1995; Giovannucci *et al*, 1998; Terry *et al*, 2002) and case–control (Benito *et al*, 1991; Benito *et al*, 1993; Tseng *et al*, 1996; Keku *et al*, 2002; Boyapati *et al*, 2004) studies. Furthermore, low serum (Bird *et al*, 1995; Kato *et al*, 1999) and erythrocyte folate concentrations (Lashner, 1993; Bird *et al*, 1995; Paspatis *et al*, 1995) have been reported to be associated with the development of adenoma or cancer.

Hypomethylation of DNA induced by a lack of folate may predispose to genetic defects associated with the development of neoplasia (Molloy *et al*, 2002). The methylation cycle maintains the level of the methyl donor *S*-adenosylmethionine (SAM) in cells. The methyl group is provided to DNA through SAM by methyltransferases, which vary according to cell type, and the product of the reaction is *S*-adenosylhomocysteine (SAH). This is immediately hydrolysed to homocysteine and adenosine. The homocysteine can then be either catabolised through cystathionine synthase (CBS) to pyruvate and used for energy or it can be used by the enzyme methionine synthase (MS) for recycling back to methionine and SAM. Methionine synthase has a requirement for a folate cofactor, 5-methyltetrahydrofolate. An inadequate supply of folate results in elevations in the plasma and cellular concentrations of homocysteine. This in turn causes an elevation of SAH, which is a potent inhibitor of all methyltransferase enzymes. Such inhibition, it is suggested, would impair the methylation of DNA, leading to changes in normal patterns of DNA methylation that regulate gene expression.

Hypomethylation at critical sites is associated with oncogene activation and tumorigenesis (Sharrard *et al*, 1992), while hypermethylation of the normally unmethylated CpG islands in the promoter regions of some tumour suppressor genes prevents transcription and promotes tumorigenesis (Issa *et al*, 1994; Hiltunen *et al*, 1997; Iacopetta *et al*, 1997).

Decreased genomic DNA methylation has been reported in colorectal tumour tissue (Feinberg and Vogelstein, 1983; Goelz *et al*, 1985) and normal-appearing colonic mucosa from individuals with cancer (Cravo *et al*, 1994). A recent study suggested that DNA hypomethylation in normal-appearing colonic mucosa is associated with low folate status and increased risk of colorectal neoplasia (Pufulete *et al*, 2003). However, no studies have investigated the relationship between DNA methylation in colonic mucosa and folate status in subjects without colorectal adenoma or cancer.

Several common polymorphisms of the remethylating enzymes methyltetrahydrofolate reductase (MTHFR), methionine synthase (MS) and cystathionine synthase (CBS) have been identified. The MTHFR 677C → T mutation is associated with increased turnover of folate and elevated plasma homocysteine concentration. Two studies suggested that leucocyte DNA is hypomethylated in individuals with low folate status and homozygous for the MTHFR 677C → T mutation (Stern *et al*, 2000; Friso *et al*, 2002). Furthermore, in a recent folate depletion–repletion study, young women carrying the MTHFR 677 TT genotype had a greater increase in leucocyte DNA methylation following repletion with folate than those who did not carry the mutation (Shelnutt *et al*, 2004). The CBS gene has a variant, 844ins68, which has been linked with decreased plasma homocysteine concentration and thus may protect against hypomethylation (Shannon *et al*, 2002). Homozygotes for the MTHFR 677C → T and MS 2756A → G polymorphisms have been found to have a lower risk of colorectal cancer (Chen *et al*, 1996; Ma *et al*, 1999), but only in individuals with adequate folate status and/or low alcohol intake. However, under conditions of low folate status, risk of colorectal neoplasia may be higher in individuals homozygous for the MTHFR 677C → T mutation (Tseng *et al*, 1996; Levine *et al*, 2000; Ulvik *et al*, 2001; Boyapati *et al*, 2004). A recent study in Japanese individuals with cancer has shown that the MTHFR 677C → T and 1298A → C mutations were linked with promoter hypermethylation in the cancer tissue (Oyama *et al*, 2004). Furthermore, patients who were homozygous for the MTHFR 677C → T mutation had significantly lower concentrations of folate in their tumour tissue (Kawakami *et al*, 2003). To date, no studies have investigated the effect of genotype on genomic DNA methylation status in healthy colonic mucosa.

The aim of the present study was to assess the influence of folate status on DNA methylation in colonic mucosa in subjects without colorectal adenoma or cancer. Common polymorphisms of the remethylating enzymes were measured to account for potential confounding effects of genotype on folate status and DNA methylation.

## MATERIALS AND METHODS

### Subjects

Subjects were patients referred for colonoscopy at the Department of Colorectal Surgery, King's College Hospital, London, UK. Exclusion criteria included a previous or current diagnosis of cancer or colorectal polyps, a strong family history of colorectal cancer or adenomatous polyposis coli, inflammatory bowel disease and current or past history of gluten-sensitive enteropathy, clinical and/or laboratory evidence of intestinal malabsorption, pregnancy, alcoholism, anaemia (haemoglobin concentration <11.5 g dl^−1^ for women and <12.0 g dl^−1^ for men), low serum B_12_ concentration (<180 ng l^−1^) and use of medication known to antagonise the metabolism of folate. Subjects were eligible for inclusion in the study if they showed no abnormality on full colonoscopy. Rectal bleeding was attributable to the presence of haemorrhoids in subjects referred for colonoscopy with this symptom. Written consent was obtained from all subjects.

Prior to the colonoscopy, a fasting venous blood sample was collected using the vacutainer technique. Blood was collected into EDTA vacutainers for full blood count, plasma homocysteine and erythrocyte folate assays and into vacutainers containing no anticoagulant for determination of serum vitamin B_12_ and folate concentrations and liver function tests. For plasma homocysteine, the vacutainers were chilled on ice and plasma was separated within 2 h of collection and frozen at −70°C. Weight and height were recorded and information on smoking habits, physical activity levels, current medication and supplement use was gathered before colonoscopy. During colonoscopy, three mucosal biopsies were removed from the rectum (about 12 cm from the anal verge) and immediately snap frozen in liquid nitrogen. All patients had prepared for the colonoscopy by taking orally administered colonic lavage solution (KleanPrep®, Norgine, Harefield, UK).

At 1 week after the colonoscopy, subjects completed a previously validated short food frequency questionnaire (Pufulete *et al*, 2002) to assess habitual intake of alcohol and folate. It was decided that the FFQ should not be administered at the time of colonoscopy, as subjects had been fasting and taking bowel preparation and were anxious about the procedure, which may have affected their response. The protocol for the study was approved by the Research Ethics Committees at King's College Hospital and King's College London.

### Laboratory methods

Fasting venous blood samples were obtained prior to the colonoscopy for determination of serum and erythrocyte folate concentrations, plasma homocysteine and MTHFR 677C → T and 1298A → C, MS 2756A → G and CBS 844ins68 polymorphisms. Rectal biopsies of normal-appearing mucosa for the determination of genomic DNA methylation were obtained during the colonoscopy.

The laboratory methods used to analyse serum and erythrocyte folate concentrations, plasma homocysteine, genomic DNA methylation and gene polymorphisms have been described in detail elsewhere (Pufulete *et al*, 2003). Genomic DNA methylation was determined in colonic mucosa using an *in vitro* methyl acceptance assay (Balaghi and Wagner, 1993). In this assay, a decrease in methyl group incorporation in DNA indicates an increase in methylation status. Within run and between run, CVs were 5.3 and 8.9%, respectively.

### Statistics

Analyses were performed using Intercooled Stata (version 6.0. for Windows, Stata Corporation, USA). Logarithmic transformations were used for erythrocyte folate, serum vitamin B_12_ and plasma homocysteine as the distribution of these variables was positively skewed. Analysis of variance was used to compare folate intake, serum and erythrocyte folate, plasma homocysteine and genomic DNA methylation for each genotype, after adjusting for gender, age, body mass index and smoking. The relationships between continuous variables were examined using the Pearson and Spearman correlation coefficients.

The relationships between markers of folate status (dietary folate intake, serum and erythrocyte folate) and each of plasma homocysteine and genomic DNA methylation in colonic mucosa were analysed by multiple linear regression with adjustment for gender, age, body mass index, smoking and genotype.

## RESULTS

A total of 68 subjects (33 men and 35 women), aged 36–78 years, were found to be eligible for the study; their details are shown in Table 1. Blood counts and serum vitamin B_12_ concentrations were all within the normal laboratory range.

Table 2 shows mean values of folate intake, serum and erythrocyte folate, plasma homocysteine concentrations and [^3^H]methyl incorporation in colonic DNA according to the genotypes, adjusted for gender, age, BMI and smoking. Allele frequencies for the MTHFR 677C → T, MTHFR 1298A → C, MS 2756A → G and CBS 844ins68 mutations were 44, 35, 40 and 25%, respectively. There was heterogeneity between the means for each of serum folate and plasma homocysteine and alleles of the MTHFR 677C → T polymorphism (*P*=0.007 and *P*=0.0002, respectively), and between [^3^H]methyl incorporation in colonic DNA and alleles of the MS 2756A → G polymorphism (*P*=0.05). The mean serum folate concentration was 45% lower and the mean plasma homocysteine concentration 110% higher in subjects homozygous for the T allele of the MTHFR 677C → T genotype than in those homozygous for the C allele.

[^3^H]methyl incorporation in colonic DNA was lower in subjects carrying the G allele for the MS 2756A → G mutation (by 18 and 28% in subjects homozygous (GG) and heterozygous (AG), respectively) than in those without the mutation (AA). There was no influence on [^3^H]methyl incorporation in colonic DNA of the MTHFR (677C → T and 1298A → C) and CBS 844ins68 genotypes.

Smokers had higher mean [^3^H]methyl incorporation in colonic DNA than nonsmokers (490 (s.d. 193) Bq *μ*g DNA^−1^ *vs* 353 (s.d. 190) Bq *μ*g DNA^−1^, *P*=0.01), and lower folate intakes (303 (s.d. 134) *μ*g day^−1^ *vs* 380 (s.d. 113) *μ*g day^−1^, *P*=0.03) and serum folate concentrations (6.9 (s.d. 3.2) *μ*g l^−1^ *vs* 8.8 (s.d. 3.4) *μ*g l^−1^, *P*=0.03).

Folate intake was positively correlated with both serum folate (*r*=0.515, *P*<0.001) and erythrocyte folate concentrations (*r*=0.473, *P*<0.001). Plasma homocysteine concentration was positively correlated with age (*r*=0.323, *P*=0.007) and negatively correlated with folate intake (*r*=−0.284, *P*=0.027), serum folate (*r*=−0.573, *P*<0.001), erythrocyte folate (*r*=−0.307, *P*=0.01) and serum vitamin B_12_ concentrations (*r*=−0.402, *P*=0.001). Multiple linear regression analysis with plasma homocysteine concentration as the dependent variable showed that gender (*P*=0.05), age (*P*=0.05), serum folate (*P*=0.001), serum vitamin B_12_ (*P*=0.002) and MTHFR 677C → T polymorphism (with higher homocysteine concentration in subjects homozygous for the T allele, *P*=0.002) were each associated with plasma homocysteine, independent of body mass index, smoking and other genotypes.

[^3^H]methyl incorporation in colonic DNA was positively correlated with age (*r*=0.260, *P*=0.03) and plasma homocysteine (*r*=0.256, *P*=0.04) and negatively correlated with serum folate (*r*=−0.311, *P*=0.01), erythrocyte folate (*r*=−0.356, *P*=0.003) and serum vitamin B_12_ concentrations (*r*=−0.218, *P*=0.08). There was no association between [^3^H]methyl incorporation in colonic DNA and alcohol intake (*r*=0.013, *P*=0.92). Multiple linear regression analysis with [^3^H]methyl incorporation in colonic DNA as the dependent variable showed a weak positive association with smoking (*P*=0.05), and weak negative associations with each of serum folate (*P*=0.07) and erythrocyte folate (*P*=0.08), independent of gender, age, body mass index and all genotypes. No other associations were noted.

## DISCUSSION

The aim of this study was to determine the contribution of folate status to genomic DNA methylation in colonic mucosa in subjects with no colorectal abnormalities. DNA methylation was determined by measuring [^3^H]methyl incorporation in DNA. Thus, an increase in [^3^H]methyl incorporation reflects a decrease in methylation. The [^3^H]methyl acceptance assay is indirect and only semiquantitative; a known limitation of this assay is that damaged DNA templates (DNA adducts, strand breaks and abasic sites) can accept methyl groups in the methylase reaction and may therefore give a false positive indication of hypomethylation. Despite this drawback, there was a trend for an association between DNA hypomethylation, smoking and folate status. It is well known that the dietary habits of smokers differ from those of nonsmokers, and in the present study the dietary intake of folate was lower in the smokers. However, the effects of smoking and folate appeared to be independent predictors of hypomethylation. A limitation of the present study is that we did not measure colonic mucosal folate concentrations. DNA methylation was not associated with alcohol intake, which is known to have negative effects on methyl metabolism and is associated with increased risk of colorectal cancer (Chen *et al*, 1996). However, median alcohol intake in this group of individuals was relatively low. A wider range of intakes would need to be considered before firm conclusions on the relationship between DNA methylation and alcohol intake can be drawn.

Common polymorphisms in remethylating enzymes were measured to adjust for possible confounding influences on DNA methylation and folate status. The frequencies of the mutated alleles for MTHFR 677C → T and 1298A → C were 44 and 35%, respectively, which were similar to those reported in other Caucasian populations: 35% for MTHFR 677C → T (Frosst *et al*, 1995; Gudnason *et al*, 1998) and 30% for MTHFR 1298A → C (van der Put *et al*, 1998). Allele frequencies for MS 2756A → G and CBS 844ins68 were somewhat higher (40 and 25%, respectively) in the present study compared with 20% (Leclerc *et al*, 1996) and 8% (Tsai *et al*, 1999), respectively, reported in other Caucasian populations. Individuals carrying the G allele for the MS 2756A → G genotype had higher genomic DNA methylation status in colonic mucosa, although DNA methylation was slightly higher in heterozygotes (AG) than in those homozygous for the G allele (GG). This finding lends some support to the observation of an inverse association between the MS 2756A → G genotype and colorectal cancer risk (Ma *et al*, 1999). Several studies have also shown that plasma homocysteine concentrations are lower in subjects carrying the G allele (Harmon *et al*, 1999; Chen *et al*, 2001). This may indicate some benefit associated with this polymorphism, although to date the functional consequences for enzyme activity of the MS 2756A → G genotype are unclear.

In the present study, we were unable to demonstrate any influence of the MTHFR 677C → T and MTHFR 1298A → C, and CBS 844ins68 genotypes on DNA methylation but the number of subjects homozygous for the two MTHFR polymorphisms or carrying the CBS 844ins68 was small. A much larger and appropriately designed study would be required to investigate the effects of common genetic variations on DNA methylation and to assess any gene–gene interactions. However, the findings of the multiple regression analysis suggest that folate status may be a stronger determinant of colonic DNA methylation than genotype. In colorectal tumours, genomic DNA hypomethylation is often associated with altered methylation patterns in CpG islands in the promoter regions of critical genes. Promoter hyper- and hypomethylation has been shown to silence tumour suppressor genes (Hiltunen *et al*, 1997) and activate oncogenes (Sharrard *et al*, 1992), respectively. Future studies should investigate the possible link between folate and CpG island methylation, particularly since there is evidence suggesting that CpG island hypermethylation in critical genes (e.g., oestrogen receptor, ER; mismatch repair, MLH1) occurs in normal colonic mucosa (Issa *et al*, 1994; Nakagawa *et al*, 2001).

In conclusion, this study suggests that folate status may be a determinant of the methylation status of genomic DNA in colonic mucosa and provides evidence for a plausible mechanism through which folate may influence risk of colorectal cancer. However, the small sample size precludes any definitive conclusions to be drawn. The findings of this pilot study require confirmation in a larger prospective cohort study.

## Figures and Tables

**Table 1:** Subject characteristics by gender

	**Men (*n*=33)**	**Women (*n*=35)**	**All (*n*=68)**
Age (year)^a^	57.6±12.1	57.2±11.9	57.4±11.9
Body mass index (kg m^−2^)^a^	26.9±4.1	26.5±4.9	26.7±4.5
Total folate intake (*μ*g day^−1^)^a^	380±137^b^	341±106^c^	359±123^d^
Alcohol intake (g day^−1^)^e^	5.0 (0.0–47.0)^b^	1.5 (0.0–38.0)^c^	3.0 (0.0–47.0)^d^
Hemoglobin (g dl^−1^)^e^	14.5 (12.2–17.2)	13.8 (11.9–16.9)	13.8 (11.9–17.2)
Serum folate (*μ*g l^−1^)^a^	8.1±3.7	8.3±3.2	8.2±3.4
Erythrocyte folate (*μ*g l^−1^)^e^	289 (143–645)	278 (110–1040)	286 (110–1040)
Serum vitamin B_12_ (ng l^−1^)^e^	444 (218–1252)	409 (201–724)	425 (201–1252)
Plasma homocysteine (*μ*mol l^−1^)^e^	11.4 (6.5–61.4)	9.6 (5.5–25.1)	9.9 (5.5–61.4)
[^3^H]methyl incorporation in colonic DNA (Bq *μ*g DNA^−1^)^a^	375±200	413±201^f^	394±200^g^
Smoking (*n* (%))			
Current smoker	12 (36)	9 (26)	21 (31)
Nonsmoker	21 (64)	26 (74)	47 (69)

a Arithmetic mean±s.d.

b (*n*=29).

c (*n*=32).

d (*n*=61).

e Median (with range in parentheses).

f (*n*=34).

g (*n*=67).

**Table 2:** Mean folate intake, serum folate, erythrocyte folate, plasma homocysteine and [^3^H]methyl incorporation in colonic DNA for all subjects and according to MTHFR, MS and CBS genotypes

		**Folate intake (*μ*g day^−1^)^a^**	**Serum folate (*μ*g l^−1^)**	**Erythrocyte folate (*μ*g l^−1^)**	**Plasma homocysteine (*μ*mol L^−1^)**	**[^3^H]methyl incorporation in colonic DNA (Bq *μ*g DNA^−1^)^b^**
	***n* (%)**	**Mean (95% CI)^c^**	**Mean (95% CI)^c^**	**Mean (95% CI)^d^**	**Mean (95% CI)^d^**	**Mean (95% CI)^c^**
All	68 (100)	359 (329–390)	8.2 (7.5–9.0)	296 (271–323)	10.9 (9.9–12.0)	394 (352–439)
						
*MTHFR 677C → T*						
CC	38 (56)	378 (337–419)	9.3 (8.3–10.3)	303 (268–343)	9.9 (8.8–11.1)	381 (321–440)
CT	24 (35)	326 (272–380)	7.4 (6.1–8.7)	279 (236–326)	10.8 (9.3–12.5)	407 (330–484)
TT	6 (9)	362 (260–464)	5.1 (2.5–7.8)	318 (230–438)	20.8 (15.4–28.3)	447 (291–603)
		*P*=*0.35*	*P*=0.007	*P*=0.66	*P*=0.0002	*P*=0.69
						
*MTHFR 1298A → C*						
AA	44 (65)	355 (315–394)	8.1 (7.1–9.2)	301 (268–337)	10.8 (9.6–12.2)	408 (352–464)
AC	22 (32)	365 (311–418)	8.3 (6.8–9.8)	291 (247–344)	11.5 (9.6–13.8)	389 (309–468)
CC	2 (3)	395 (226–565)	9.9 (5.1–14.7)	244 (143–417)	7.6 (4.3–13.4)	209 (47–465)
		*P*=0.91	*P*=0.78	*P*=0.75	*P*=0.37	*P*=0.33
						
*MS 2756A → G*						
AA	41 (60)	354 (315–393)	8.4 (7.3–9.4)	283 (252–318)	10.7 (9.4–12.2)	438 (383–492)
AG	19 (28)	359 (302–415)	8.0 (6.5–9.6)	322 (271–382)	11.5 (9.5–13.9)	317 (233–400)
GG	8 (12)	390 (296–485)	8.2 (5.7–10.7)	304 (230–401)	10.6 (7.8–14.3)	359 (230–489)
		*P*=0.86	*P*=0.94	*P*=0.47	*P*=0.81	*P*=0.05
						
*CBS 844ins68*						
Wild type	51 (75)	355 (321–390)	8.3 (7.3–9.2)	286 (258–317)	10.8 (9.6–12.0)	404 (352–455)
844ins68	17 (25)	372 (310–435)	8.2 (6.5–9.9)	328 (273–395)	11.4 (9.3–14.0)	372 (282–463)
		*P*=0.60	*P*=0.96	*P*=0.22	*P*=0.61	*P*=0.57

MTHFR=methylenetetrahydrofolate reductase; MS=methionine synthase; CBS=cystathionine synthase.

a (*n*=61).

b (*n*=67).

c Arithmetic or

d Geometric means (with 95% CI in parentheses) adjusted for gender, age, BMI and smoking. The *P*-values relate to tests of heterogeneity between the means arising from the relevant *F*-test statistic in the analysis of variance table.
